# Rare Cause of Seizures, Renal Failure, and Gangrene in an 83-Year-Old Diabetic Male

**DOI:** 10.1155/2013/523865

**Published:** 2013-06-23

**Authors:** Stalin Viswanathan, Kandan Balamurugesan

**Affiliations:** Indira Gandhi Medical College, Kadhiramam, Pondicherry 605009, India

## Abstract

We report an 83-year-old diabetic male who presented with acute-onset renal failure, seizures, psychosis, pneumonia, and right foot gangrene. Investigations revealed thrombocytopenia, CSF lymphocytosis, ANA and dsDNA positivity, hypocomplementemia, and pneumonitis following which he was treated with pulse methylprednisolone. He was treated for *Pseudomonas*-related ventilator-associated pneumonia, candiduria, and *E. coli*-related bedsore infection prior to discharge. He was discharged at request and died 17 days later due to a respiratory infection.

## 1. Introduction

Onset of systemic lupus erythematosus (SLE) after the age of 50 (late-onset SLE) constitutes 6–18% of the lupus population [1]. Most cases of lupus over 65 years have been described as case reports. Renal failure is the initial presentation only in 25% patients of SLE [2]. Neuropsychiatric SLE (NSLE) in the elderly is very rare. Infections, malignancies, and atherosclerotic disease account for most deaths in SLE patients [3]. Here we describe an 83-year-old diabetic who presented with acute-onset seizures, psychosis, pneumonitis, foot gangrene, and renal failure and improved with immunosuppressive therapy for SLE but succumbed to another respiratory infection 17 days after discharge from hospital.

## 2. Case

This 83-year-old diabetic of 10 years' duration (on metformin 750 mg OD) was brought from another hospital by his relatives for mechanical ventilation. Fifteen days prior, he had complained of fatigue and anorexia and was admitted in a local nursing home where he was told to have early renal failure (creatinine 202 *μ*mol/L). Four days later he had had a generalized tonic-clonic seizure for which he was taken to the referring hospital for management. Computed tomography (CT) of brain was normal and the patient was commenced on phenytoin; he was uncooperative for magnetic resonance imaging (MRI). He had developed acute psychosis and delirium in hospital and was managed with risperidone. Three days later he was intubated for altered sensorium and respiratory distress following acute cough, breathlessness, and fever. He was mechanically ventilated and administered ceftriaxone and metronidazole; his seizures remained under control but altered sensorium persisted. During his stay in intensive care, he developed discoloration of his right foot and warfarin had been initiated. His renal parameters had continued to worsen (creatinine 350 *μ*mol/L) and his relatives requested discharge and brought him to our hospital.

**Figure 1:**
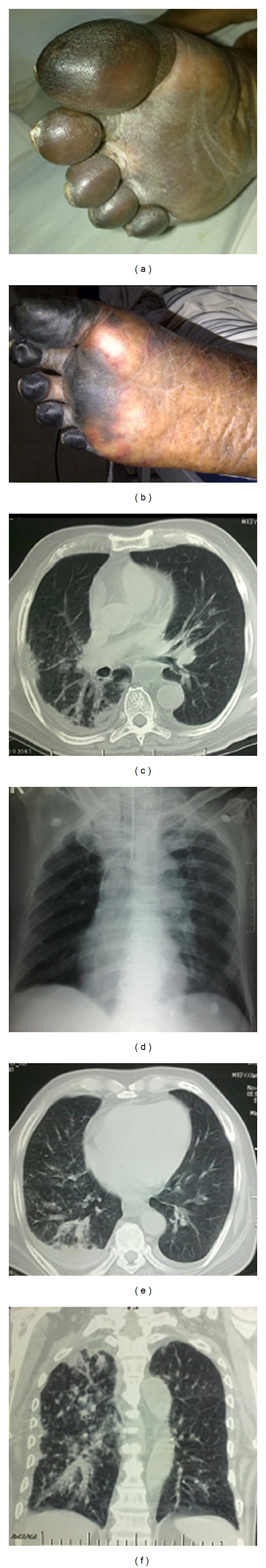
(a) Right foot discoloration at admission. (b) Right forefoot dry gangrene on day 4. (c) CT chest on day 2 showing airway dilatation (right predominant), patchy infiltrates, and ground glassing. (d) Chest radiograph on day 4 of admission shows clearing of infiltrates in the right side. (e) CT chest revealing right-sided pleural effusion, reticular infiltrates, bilateral ground glassing, and tree-in-bud appearance. (f) CT chest shows reticulonodular infiltrates in the entire right lung and ground glassing in left lung.

He was a cigar smoker (>40 years) and drank occasionally. He had had a left hip fracture six years ago which had been treated conservatively. On admission, his pulse was 104 bpm, BP 92/60 mm Hg, respiratory rate 42 breaths/min with SpO_2_ 92% on FiO2 of 100, temperature 99°F, and central venous pressure (CVP) was 6 cm. Chest examination revealed left-sided coarse crackles. The Glasgow Coma Scale (GCS) of 2T/15, bilaterally 3 mm sluggishly reacting pupils, generalized hypotonia, and areflexia were observed on neurological examination. There was no papilledema. He had dry gangrene of right foot (Figure 1) with weak right popliteal pulse. Investigations are listed in Table 1. Doppler of right lower limb and echocardiography were normal. Pending cultures, he was initiated on piperacillin-tazobactam and levofloxacin, along with subcutaneous heparin, warfarin, and pentoxifylline. CT chest (day2) showed bilateral pleural thickening, bilateral ground glassing (right ≫ left), airway dilatation, and reticulo-nodular infiltrates (right predominant) with minimal pleural effusion (Figure 1). In view of seizures, psychosis, thrombocytopenia, renal failure, pneumonitis, ANA, and dsDNA positivity, a diagnosis of systemic lupus was made and pulse methyl prednisolone (1 g × 3 days) was initiated on day 3, followed by oral steroids (60 mg). Tracheal aspirate grew *Pseudomonas aeruginosa* and imipenem was administered on day 5 for probable ventilator-associated pneumonia. Weaning was done on 8th day of admission. Fluconazole 300 mg/day was administered for persisting candiduria. Hypernatremia was managed with dextrose saline, while sugars were controlled with infusion of regular insulin. By day 12, his power had improved to 3/5 in all limbs; he occasionally spoke a few words to his relatives but continued to be extremely afraid of hospital personnel. MRI and nerve conduction studies could not be performed due to poor cooperation. He developed an infected gluteal bedsore (*E. coli*) that necessitated amikacin. We acceded to his son's request to be discharged to home with modified doses of intramuscular amikacin therapy, twice-daily premixed insulin, warfarin, phenytoin, risperidone, clonezepam, and oral prednisolone 40 mg/day. Seventeen days later he succumbed to another respiratory infection.

## 3. Discussion

The 9 : 1 female predominance in SLE decreases prior to puberty and late in life [4]. Female : male ratio in late-onset SLE is about 5 : 1 [5], while another study showed a ratio of 1 : 1.1 when the age of onset was >65 years [6]. Four to 18% of cases from reported series are male [7]. In a Hong Kong study, the mean age of late-onset SLE was 62 years and onset was generally insidious [1]. Insidious onset of disease and lower index of suspicion lead to delayed diagnosis in the elderly. A study of 39 Indian male SLE subjects showed only one patient with late-onset SLE [8]. 

It is generally agreed that SLE in the elderly is a mild disease [9]. Prevalence of organ involvement in males depends upon the ethnic population being studied, study setting (tertiary versus primary), selection criteria of female controls, and sample size of male subjects [10]. Fever, fatigue, and weight loss are common symptoms in elderly SLE patients [5]. Serositis, muscle pains, and arthritis are more common in this age group as are secondary Sjögren's syndrome but with a lower incidence of cutaneous manifestations and Raynaud's phenomenon [9]. Males in a Thai study tended to have a shorter duration of symptoms prior to presentation, with alopecia, arthralgia and Raynaud's phenomenon being less common [10]. Psychosis, hypocomplementemia, and diffuse proliferative glomerulonephritis (DPGN) were less common in Indians [8], while renal disease and vascular thrombosis were common among Latin American males [11]. Rheumatoid arthritis, polymyalgia rheumatic, and sicca syndromes are close differentials of SLE in the elderly [5]. Late-onset lupus may have fewer major organ involvement and fewer major relapses [12].

 Among patients with SLE, 60% of adults develop kidney disease [2]. SLE prevalence in India was low at 3.2/100000 population [13]; contrastingly, renal involvement among Indian SLE patients was the highest in the world [13]. Neuropsychiatric manifestations are similar in the young and the elderly [9]. Neuropsychiatric SLE (NSLE) at presentation in the elderly population has been described only as case reports. Similar to our case, seizures, coma, and pneumonia have been reported in a 72-year-old lady who had pneumonitis, hypocomplementemia, elevated fibrinogen, and FDP but with normal renal function and negative dsDNA [14]. Presence of NSLE is generally associated with a poor prognosis [9]. NSLE can be either focal (stroke, neuropathy, and transient ischemic attack) or diffuse (confusion, dementia, and psychosis) or can present with seizures (partial or generalized) [9]. Seizures are reported in 15 to 30% of patients with SLE [9]. Cognitive impairment may be the initial manifestation of SLE in the elderly [5]. The neurological manifestations seen in Indian studies were cerebrovascular accidents, myeloradiculopathies, movement disorders, seizures, coma, and psychosis [13]. Lower numbers of Raynaud and NSLE were seen in the South Indian population [13]. Our patient's cognition did not improve completely at time of discharge. His respiratory symptoms could have also been contributed by diabetes-related pneumonia or seizure-related aspiration pneumonia but his chest radiograph (Figure 1) cleared on the 4th day of admission (after two methyl prednisolone pulses). The cause of foot gangrene could not be pinpointed. Antiphospholipid antibody testing was unavailable in our hospital. Since warfarin had been initiated prior to admission, protein C and protein S were not done. High levels of D-dimer and FDP like in our patient may indicate vascular involvement due to emboli and/or inflammation [14]. It is possible that diabetes, old age, smoking, sepsis, and SLE predisposed him towards thrombosis.

Age did not affect serological findings in a study [5]. False positive ANA can be seen in the elderly [5]. Prevalence of dsDNA positivity and hypocomplementemia may be lower [5, 9] and complement levels are inversely proportional to the age [9]. Our patient's C3 levels were borderline low, while ANA and dsDNA were positive. Anti-Ro and anti-La antibodies can be useful adjuncts in the elderly when dsDNA levels are less frequently positive [9]. The American Rheumatological Association (ARA) criteria may be too strict in the elderly population with NSLE and hence more attention is given to serology in the elderly [15]. Also, patients may not satisfy the current ARA classification criteria at presentation and hence diagnosis may be uncertain; they would need a longer duration of followup for the diagnosis to be made [12].

CNS disease and renal involvement contributed towards mortality [3]. Nonrenal factors like younger age, male sex, and hematological complications like thrombocytopenia were prognostic factors in lupus nephritis [2]. Cardiovascular disease and infections are common causes of mortality in the elderly [5]. In a retrospective Indian study, mortality in SLE patients was due to disease activity, infection, or both [3]. Hospital-acquired Gram-negative septicaemia contributed most in this study. Our patient had disease activity along with candiduria and *Pseudomonas*-related pneumonia which improved prior to discharge, but our patient finally succumbed to an infection. Septic shock due to high-dose immunosuppressants was the major cause of morality in older-onset SLE.

In conclusion, we report an elderly male diabetic with late-onset NSLE, gangrene, and sepsis (respiratory and urinary) that improved with immunosuppressant therapy. Systemic lupus erythematosus is an autoimmune disease involving women of childbearing age with highly variable clinical presentations and with 10% of cases occurring in older patients. Arthritis, fever, serositis, Raynaud's syndrome, lung disease, neuropsychiatric symptoms, positive antinuclear antibody tests, positive rheumatoid factor, positive anti-Ro/Sjögren's syndrome (SS) A, and positive anti-La/SSB are more common in patients with elderly-onset lupus. Autoimmune diseases are very rare in elderly males and need to be considered in the differential diagnoses when confronted with multisystem disease even in the presence of diabetes and systemic sepsis. The diagnosis of elderly-onset lupus is often delayed for several months because of insidious onset and similarity to other more common disorders.

## Figures and Tables

**Table 1:** Lab investigations of patient.

Day of admission	1	4	5	7	10	16	18
Urea (2.5–7.1 mmol/L)	67.8	61.7	47.1	38.7	33.2	26.4	21.1
Creatinine (44–80 *μ*mol/L)	616	422	360	281	290	202	167
HbA1c (5.7–6.5%)	7.9						
Bil total (1.7–6.8 *μ*mol/L)	1.53						
Bil dir (3.4–15.2 *μ*mol/L)	8.5						
SGOT (0.20–0.65 *μ*kat/L)	1.5				1.4		
SGPT (0.12–0.70 *μ*kat/L)	0.87				0.92		
ALP (0.56–1.63 *μ*kat/L)	3.88				9.02		
Protein (67–86 g/L)	63						
Albumin (40–50 g/L)	18	21	22	22			
GGT (0.15–0.99 *μ*kat/L)	4.48						
K^+^ (3.5–5.0 mmol/L)	5.6	3.3	3.9	3.2	145	137	138
Na^+^ (136–146 mmol/L)	155	151	150	143	5.7	3.8	3.2
Calcium (2.2–2.6 mmol/L)	2.2						
Mg (0.62–0.95 mmol/L)	0.78						
Pi (0.81–1.4 mmol/L)	1.45						
LDH (114–240 IU/L)	506				269		
CK (25–200 U/L)	363				103		

*Urine *							
Spot K^+^ (25–120 mEq/L)	30.5						
Spot Na^+^ (40–220 mEq/L)	116						
Bence-Jones	Negative						
Myoglobin	Negative						
Heme	Negative						
Eosinophils	Negative						
Culture	3 organisms						
Stool occult blood	Negative						
Endotracheal asp AFB	Negative						
Endotracheal asp culture	Pseudomonas						
Hb (130–160 g/L)	81						
TC (3.50–9 × 10^9^/L)	10.2						
Neutrophilia (%)	81						
Plat (165–415 × 10^9^/L)	100						
MCV (79–93.3 fL)	77						
MCH (26.7–31.9 pg)	27.8						
MCHC (323–359 g/L)	358						
Reticulocyte (%)				0.5			
INR				1.3			
aPTT (control 25.1 s)				40			
D dimer (200 ng/mL)				3200			
FDP	Positive						
Blood cultures	Sterile						
dsDNA (<20)	28						
ANA (<1.0)	1.8						
cANCA	Negative						
C3 (0.83–1.77 g/L)	0.78						
C4 (0.16–0.47 g/L)	0.20						
Cortisol (5–25 *μ*g/dL)	26.1						
Ferritin (28–397 ng/L)	1328						
Direct Coombs	Negative						

*Cerebrospinal fluid *							
Cells	103						
Sugar (mg)	115						
Protein (<60 mg)	39						
Lymphocytes (<5)	100%						
ADA	1.0						
AFB	Negative						
Gram	Negative						
India ink	Negative						

HbA1c: glycated haemoglobin; bil: bilirubin; dir: direct; SGOT: serum glutamic oxaloacetic transaminase; SGPT: serum glutamic pyruvate transaminase; ALP: alkaline phosphatase; GGT: gamma-glutamyl transferase; K^+^: potassium; Na^+^: sodium; Mg: magnesium; Pi-inorganic phosphate; LDH: lactate dehydrogenase; CK: creatine kinase; Hb: haemoglobin; TC: total cells; plat: platelets; MCV: mean corpuscular volume; MCH: mean corpuscular haemoglobin; MCHC: mean corpuscular haemoglobin concentration; INR: international normalized ratio; aPTT: activated partial thromboplastin time; FDP: fibrinogen degradation products; dsDNA: double-stranded DNA; ANA: antinuclear antibody; c-ANCA: antineutrophil cytoplasmic autoantibody; C3: complement C3; C4: complement C4; ADA: adenosine deaminase; AFB: acid-fast bacilli; asp: aspirate.
